# Integrated Confocal and Scanning Probe Microscopy for Biomedical Research

**DOI:** 10.1100/tsw.2006.269

**Published:** 2006-12-15

**Authors:** B.J. Haupt, A.E. Pelling, M.A. Horton

**Affiliations:** Rayne Institute, Department of Medicine, University College London, London WC1E 6JJ, UK

**Keywords:** atomic force microscopy (AFM), confocal microscopy, fluorescence microscopy, cell biology, nanomechanics, mechanotransduction, mechanobiology

## Abstract

Atomic force microscopy (AFM) continues to be developed, not only in design, but also in application. The new focus of using AFM is changing from pure material to biomedical studies. More frequently, it is being used in combination with other optical imaging methods, such as confocal laser scanning microscopy (CLSM) and fluorescent imaging, to provide a more comprehensive understanding of biological systems. To date, AFM has been used increasingly as a precise micromanipulator, probing and altering the mechanobiological characteristics of living cells and tissues, in order to examine specific, receptor-ligand interactions, material properties, and cell behavior. In this review, we discuss the development of this new hybrid AFM, current research, and potential applications in diagnosis and the detection of disease.

## INTRODUCTION

In recent years, atomic force microscopy (AFM) has been increasingly used to address problems of biomedical relevance. Originally developed by Binnig et al. in 1986[1] to study the material and topographic features of nonconductive surfaces, it has now become a popular tool in the study of biological samples[2]. It is capable of imaging cells at high resolution[3,4,5,6,7,8], measuring the forces involved in intermolecular bonds[3,9,10,11], and investigating the mechanical properties of biological materials[5,6,12,13,14,15,16,17,18,19,20,21,22,23,24]. AFM has the advantage of being able to operate in air and fluid under physiological conditions, which has allowed biologically relevant, force spectroscopy studies of single biomolecule binding events[10,13] and a wide range of applications in cell biology, such as studying cell-surface morphology[6,7,8,25,26], the cytoskeleton[5,6,17,18,19,20,22,23, 24,27,28,29] and organelles[6,29,30,31], cell movement[16,31,32,33], and cell-matrix or cell-cell interaction forces[3,9,34,35,36] resulting from plasma membrane receptor binding[3,9,10,11,37].

In biomedical studies, AFM can be used as a micromanipulator, applying precise pico to nano Newton forces[3,14,35,38]. As an imaging tool of biological samples, AFM cannot compete with the speed at which traditional microscopy tools, such as confocal laser scanning microscopy (CLSM) and electron microscopy (EM), capture an image. However, its main advantage in biomedicine is the ability to probe and alter the mechanobiological characteristics of living cells and tissues in order to examine specific, receptor-ligand interactions, material properties, and cell behavior. The new focus of using AFM to study biological samples is to combine it with optical imaging methods, such as CLSM and fluorescent imaging[5,6,7,8,9,12,13,14,17,18,19,20,24,26,29,30,37,38,39,40,41,42,43]. This would then allow the investigation of mechanotransduction/sensation pathways[12,14,18] as well as traditional imaging applications, such as the comparison to topographical features to the cell architecture[5,6,7,8,12,13, 14,17,19,26,29,39,40].

In this review, we show how the AFM has currently undergone a transformation from a surface science tool to a biological tool by integration with optical techniques in combination with its ability to act as a nanoscale force transducer/actuator and imaging device. We will discuss the advantages of such combinations, current research [7, 8, 10-14, 18, 24, 26, 37, 38, 40-43], the potential medical applications of AFM in diagnosis and detection of disease or ageing [44, 45], development of new and novel devices [38, 41-43], and highly specific drug studies [5, 6, 12, 19, 22-24].

## PRINCIPLES OF AFM AND INTEGRATION WITH OTHER OPTICAL TECHNIQUES

Imaging of a surface with AFM involves a microfabricated cantilever with a very small tip (with a contact area of only a few square nanometers) that is raster scanned above the surface of a sample (see Fig. 1). The movement of the cantilever is controlled by a *x,y,z*-piezoelectric ceramic tube that moves the cantilever, and a laser beam that is reflected off the back of the cantilever onto a quadrant photodiode that measures the cantilever deflection. A feedback loop linking the current applies the piezo and the detector enables precise control of the positioning of the cantilever and the force applied to the sample[39,46].

Several different modes of operation have been developed for AFM and have been reviewed by Hansma et al.[46]. Contact mode imaging involves the cantilever scanning a sample at a constant applied force, where the tip remains in constant contact with the sample surface. In this mode, the cantilever is moved up and down in the *z*-direction to remain in contact and, from this, a topographical image is obtained. Noncontact mode is another imaging mode that involves rapidly oscillating the cantilever in the *z*-direction, either mechanically (Tapping mode) or magnetically (MAC mode) above the surface while scanning. As the oscillating cantilever approaches the surface, its amplitude is decreased and it is this that is used as the feedback for imaging. This imaging mode is far less damaging to soft samples such as cells and tissues[4]. The last mode we shall describe is force mode from which force-distance measurements are made. In this mode, the cantilever is moved only in the *z*-direction towards and away from the surface with the deflection ($z$) being constantly measured (see Fig. 2). With knowledge of the spring constant ($k$) of the cantilever, it is possible to calculate the force ($F$) at any given deflection through Hookes Law ($F = -kz$). This mode can be used to study single ligand-receptor binding forces[3,9] or determine the material properties of biological samples[5,6,12,13,14,15,16,17,18,19,20,21,22,23,24].

### Measuring Receptor-Ligand Binding Events

With AFM, it has been possible to quantify receptor-ligand binding forces, typically in the range of 15–250 pN for protein interactions and up to 220 nN for cellular binding forces[9,10,11,34,35,36,37, 47,48,49,50,51,52]. To be able to study single receptor-ligand events requires that the tip of the AFM be "functionalized" by attaching a specific interactive molecule (either the "receptor" or the "ligand") that will act as a probe to detect binding events on a surface, such as the plasma membrane of a cell. The attached molecule can either be a protein, such as an antibody, or a protein ligand. The two most common techniques developed to achieve functionalization are (1) chemisorption of a cross-linker molecule to the tip, such as polyethylene glycol (PEG), which is covalently bound to the ligand[9] or (2) covalently binding the molecule directly to the tip[47], typically through a gold-sulfur bond commonly employed during the manufacture of self-assembled monolayers[53]. Force-distance curves can then be measured between the

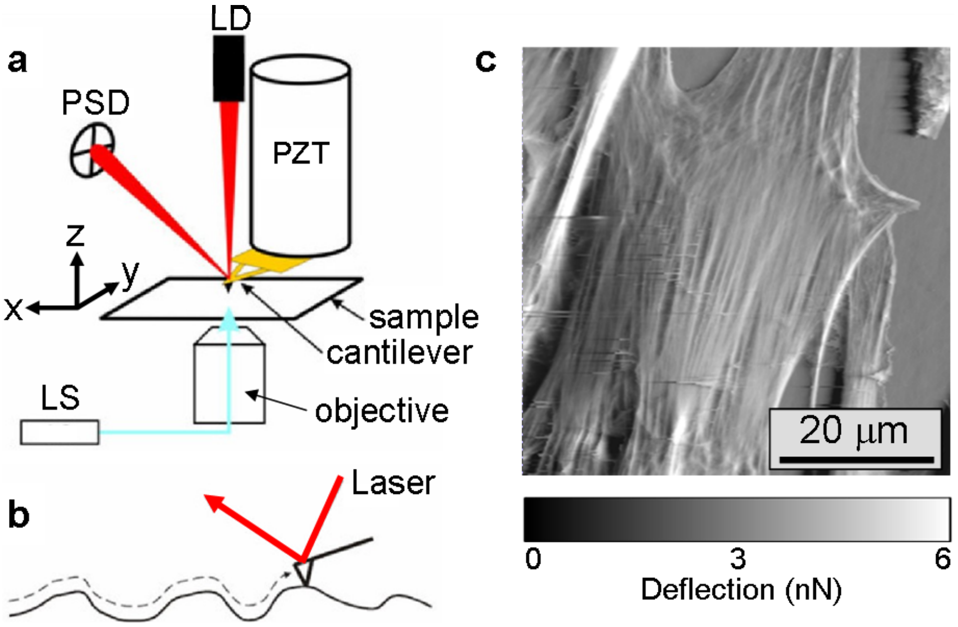

**FIGURE 1.** (a) The basic AFM setup involves an optical beam deflection design that records the deflection of a typical, commercially available, gold-coated silicon nitride AFM cantilever as it moves in response to force or topographical features. The cantilever is mounted on a piezoelectric tube scanner (PZT), which is used to raster scan the tip in the x-y directions over the surface. As it traces out the topography of the substrate, a laser diode (LD) is focused onto the back of the AFM cantilever and the beam is reflected into a position-sensitive diode (PSD). This allows the computer to record the deflection of the cantilever as it traces out the topographical features of the surface (b). The AFM can be mounted on an inverted optical microscope with phase-contrast optics and illuminated with a light source (LS) to allow simultaneous fluorescence microscopy. In another embodiment, a confocal laser scanning microscope can be integrated into the setup, allowing for simultaneous confocal microscopy. After scanning a surface, the topographical features can be reconstructed into 2D or 3D images. In (c), a 2D AFM image of a living human fibroblast cell is shown in which F-actin stress fibers are clearly visible (image obtained using the JPK Nanowizard, courtesy of A.E. Pelling, F. Veraitch, C. Chu, C. Mason, and M.A. Horton, UCL).

functionalized tip and a surface containing the binding partner. A schematic of a force-distance curve of a cell receptor-ligand event obtained using AFM is shown in Fig. 2. This technique has been commonly employed in live cell studies to receptors located in the cell membrane[9,10,35,36].

## Measuring Mechanical Properties of a Cell

AFM can be used to measure material properties of a cell in force mode, such as stiffness, plasticity, and viscoelasticity. The effects of induced cytoskeletal changes on these properties has also been studied in an attempt to elucidate the role of each cytoskeletal element[5,6,12,19,22,23,24]. By analyzing the force-distance curve of an AFM tip indenting a cell at a particular location and applying the theory of indentation, the elastic modulus can be determined[15,54]. Force-volume imaging is a technique in which traditional raster scan imaging and force-curve measurements are combined. This involves moving the tip in a raster fashion across a surface and measuring a force curve at specific locations in the image area. Typically, one will obtain a 2D array of force curves over the surface, which provides a partial map of material properties, such as elasticity. A common application is in the measurement of the spatial elasticity of a living cell[5,6,12,13,14,15,16,17,18,19,20,21,22,23,24], of particular importance is the ability of this method to measure temporal changes in elasticity as a cell undergoes physiological processes[16,19,21].

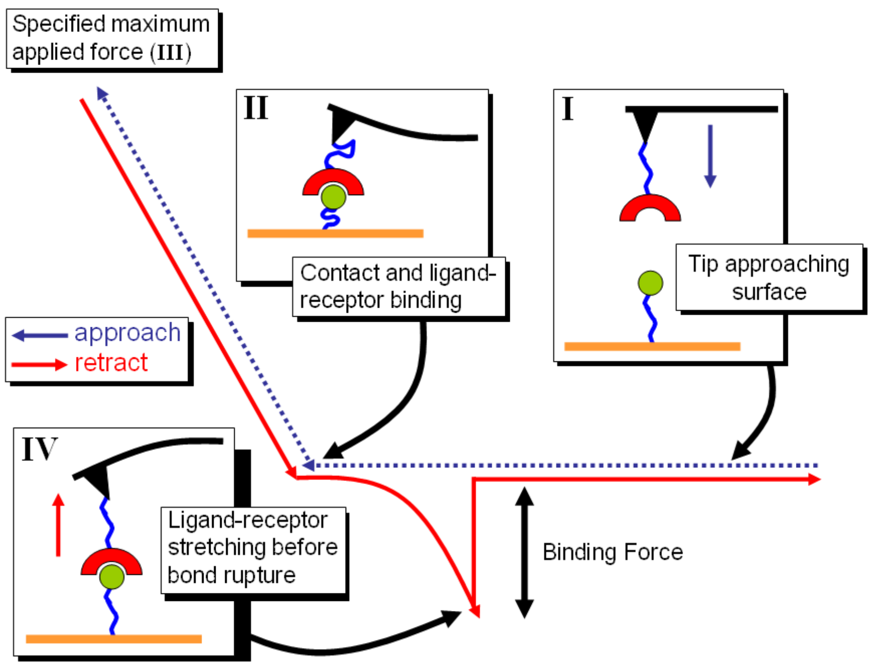

**FIGURE 2.** The anatomy of an ideal force curve measured with AFM. Initially (I), the AFM cantilever is at rest away from the surface. In this example, the cantilever and surface have been modified with a receptor and ligand, respectively. The cantilever is then lowered (blue dotted line) and will eventually come into contact with the surface (II). At this point, the receptor and ligand are in close proximity and, as the cantilever continues to press against the surface, the pair will bind while the cantilever is deflected upwards. An experimentally selected maximum applied force (III) will determine the total force that must be applied before retracting the cantilever (red line). As the cantilever is moved away from the surface, the forces holding the receptor-ligand pair together will deflect the cantilever downwards until some critical value when the molecules are stretched to their maximum (IV). When the downward force overcomes the molecular interaction, the tip will deflect upwards to its initial resting state. The difference in force between the stretched and resting state provides a measure of the binding strength of the molecular interaction. Further insights can be obtained from force curves by fitting mathematical models that allow the determination of mechanical quantities, such as the elasticity of the molecules that were stretched. Force curves can also be measured on living cells, allowing the detection of ligand-receptor binding events on the cell surface or determination of the local elasticity/viscoelasticity of the plasma membrane.

## Combining AFM and Optical Techniques

Combing AFM with other optical techniques allows for a more comprehensive study of biological systems. Previously, studies have been carried out in parallel, where the same sample is first studied on one instrument (such as the AFM) and then transferred to a second instrument (such as a fluorescence microscope)[6,9,17,20,21,29,37,39]. This transfer from one instrument to another makes it extremely difficult to study the same area, and it is nontrivial to correlate information gained through the secondary technique to measurements of manipulations conducted with AFM. In addition, mechanotransduction experiments in which the AFM tip is used to stimulate biological responses through the application of force requires that the measurements be performed simultaneously[12,14,18]. To ensure that the sample is being probed in precisely the same area, it has become necessary to integrate multiple microscopies into a single unit to perform either simultaneous or sequential studies[3,5,7,8,11,12,13,14,18,19,26,38,40, 41,42,43,55,56,57]. The hybrid AFM-confocal microscope consists of an AFM being fitted onto a modified inverted microscope, and allows the tip to be aligned into a desired position independent of the microscope stage and the sample holder (Fig. 1)[3,5,7,8,11,13,18,19,26,38,40,41,42,43,55,56,57]. In this configuration, it is possible to perform simultaneous AFM measurements on fixed or living cells, under physiological conditions, with light, DIC, and phase-contrast microscopies, video capture, total internal reflection fluorescence (TIRF) microscopy, fluorescence and CLSM.

## BIOMEDICAL STUDIES: THE STORY SO FAR

The field of combined confocal AFM (CC-AFM) and combined fluorescence AFM (CF-AFM) is still very much in its infancy. The majority of AFM studies on cell biology, mechanics, or function have either employed "side-by-side" or simultaneous approaches. Side-by-side experiments are by far the most common in which AFM measurements are performed independently or in parallel with optical/fluorescence techniques[6,7,8,9,17,20,21,26,29,37,39]. Simultaneous experiments are often performed on living cells and require AFM and optical/fluorescence measurements to be measured at the same time[3,11,12,14,18,38,41,42,43]. Confocal measurements of calcium release in response to mechanical stimulation with an AFM tip is an example of such an experiment[12,14,18]. The major limitation to the advance of simultaneous CC/CF-AFM has typically been the need for fixation to fluorescently label cells/molecules of interest and/or the long imaging times required to obtain AFM force maps or topography images (20–30 min). However, it has become clear that in both cases, these issues represent severe limitations.

It is known that fixing cells before or after AFM imaging/mechanical measurements results in changes of the mechanical and topographical properties of a cell[39]. On the other hand, studying live cells with AFM often requires long imaging times (~20–30 min) to produce topography or force maps in order to compare to a fluorescent image. The long imaging times are an important consideration because biological time constants occur in the millisecond to minute time scales (e.g., cytoskeletal dynamics[58,59,60,61,62,63]). Although these limitations are issues to be considered carefully, several novel side-by-side and simultaneous experiments have been published that have used AFM to reveal important and biologically significant phenomena that are inherent in governing the mechanical properties and responses of living cells.

## SIDE-BY-SIDE STUDIES

Early work that utilized fluorescence measurements in combination with AFM were typically concerned with the underlying basis for the mechanical rigidity of the plasma membrane[5,19,20]. One of the major advantages of AFM in cell biology is the ability to apply and measure mechanical properties of living cells locally. Several physical models exist that allow the local elasticity or Young's Modulus of the cell to be extracted from force curves measured on cell membranes[15,54]. The most commonly employed is a modification of the original Hertz model for the indentation of a homogenous elastic substrate with an indenter of well-defined geometry[13,14,15,16,17,19,20,21,22]. Either through force-volume imaging[13, 15,16,19,20,21,22] or stationary force-curve measurements on a single spot of the cell[5,6,17,23], it has been shown that internal structures, such as the underlying cytoskeleton, govern the observed stiffness of the cell membrane.

Force-volume imaging has been carried out on living cells which were subsequently fixed and imaged with fluorescence microscopy allowing researchers to show that F-actin filaments correspond directly to filamentous regions of high elasticity [5, 19, 20]. Furthermore, treatment of living cells with anticytoskeletal drugs has also shown that the dynamics of depolymerization directly affect the observed stiffness of the cell membrane[5,6,12,19,22,23,24]. Conflicting reports on the relative roles of actin, microtubules, and intermediate filaments do exist[19,23,24] and it is becoming clear that the roles of these structures in governing the local nanomechanical properties of the cell membrane are highly dependent on cell type and physiological conditions. Furthermore, AFM is also capable of sampling mechanical properties at different depths inside the cell where cytoskeletal filaments often play different mechanical roles than at the plasma membrane[24].

## SIMULTANEOUS STUDIES

Advances in the technique of AFM, in conjunction with optical techniques, have made it possible to study the physical and physiological properties of living cells at the nanoscale. Increasingly, CC-AFM and CF-AFM are being used to look at cell structure, from individual molecules to entire cells, as well as cell function in relation to their behavior, cell signaling, and disease. The use of AFM is also changing, from focusing only on surface measurements to being used as a micromanipulator, microindentor, and nanotool.

Mechanotransduction studies using AFM have been performed on osteoblasts[12,14] and myoblasts[18]. The work on osteoblasts enabled the study of cellular responses to mechanical stimuli, intracellular calcium responses, and how these responses were modulated by anticytoskeletal drugs. From this, a model was proposed linking single cell responses to whole bone adaptation. Stretch activated channels in myoblasts were studied to investigate and elucidate the role of sphingosine 1-phosphate (S1P), a lysophospholipid. By using the AFM probe to stimulate stress fibers in a living cell mechanically, the influx of Ca2+ ions could be altered. The findings of this work suggested that S1P affects skeletal muscle development through increased Ca2+ and actin cytoskeleton reorganization.

New and novel use has been made of AFM in the past few years for nanoscale surgery on living cells[38,41,42,43]. Conventional silicon tips were sharpened to ultrathin needles using a focus ion beam. By using CC-AFM, it was shown that nanoneedles are able to penetrate a cell plasma and nuclear membrane, and can be removed without causing lasting damage to the plasma membrane and nucleus. In addition, the authors were able to penetrate the plasma membrane with the sharpened tip while simultaneously imaging the process of tip insertion with CLSM. Through modification of the needle’s surface, various molecules, such as proteins, nucleic acids, DNA, and pharmacological agents, could be directly injected in to a single cell for highly specific therapeutic treatment and gene therapy. Furthermore, in similar work, an unsharpened tip was employed to extract mRNA from the cytoplasm, which was subsequently amplified[64]. This work revealed that the cells were not damaged or destroyed during the procedure and quantitatively measured changes in $\beta$-actin gene expression in response to serum within an individual living cell.

## CONCLUSIONS AND OUTLOOK

The future outlook of CC-AFM and CF-AFM, and their application to biological and medical studies, is bright. Further developments of the instrument will allow for even greater precision in the manipulation of molecules and cells. This, in conjunction with the greater demand for new immunoflourescent dyes for live cell imaging, will allow more and more biological processes to be investigated. Possibilities include single cell mechanotransduction of cardiomyocytes (see Fig. 3), the study of specific cellular structures such as the primary cilia (see Fig. 4) in relation to cell behavior and disease, and biological material studies for development as biocompatible materials. New and more novel uses will be found for AFM as a nanotool as the field grows.

In this review, we have discussed the many applications of integrated confocal and AFM for biomedical research. Future work will see this technique become more applied in the field of medicine with its development as a novel device. However, limitations with its use for imaging soft biological samples may see it used more as a micromanipulator, rather than as an imaging tool. AFM has already been put to use for highly specific drug studies, the study of numerous cell processes, as well as the mechanical properties of healthy and diseased cells. The challenge will be its modification and development as a diagnostic tool for the early detection of disease.

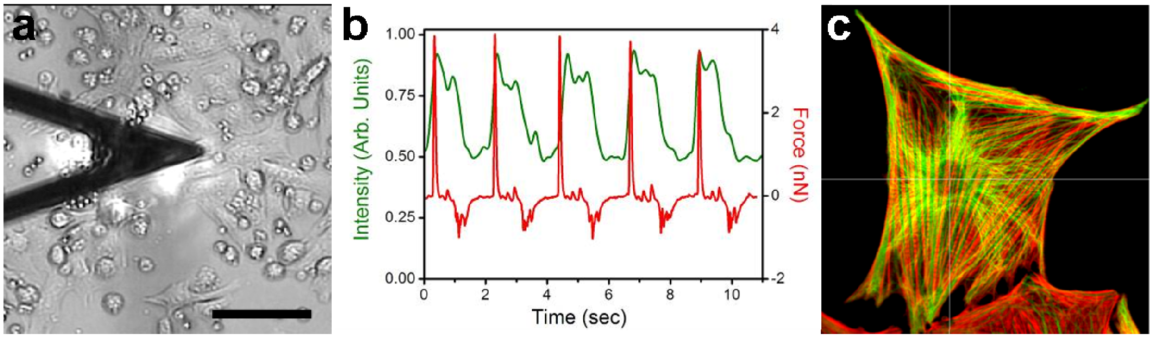

**FIGURE 3.** (a) A phase-contrast image shows an AFM tip in contact with a layer of primary, neonatal rat cardiomyocyte cells. The cells can be maintained at 37ºC in growth medium while measuring their nanomechanical contraction dynamics (scale bar = 100 µm). (b) Cells can be loaded with the calcium sensitive dye Fluo-4, while simultaneous fluorescence-AFM measurements reveal the correlation between nanomechanical contractions and calcium release. (c) In a "side-by-side" experiment, the cells measured in (a) can be fixed and imaged with CLSM (image is 126 $\mu m^2$). The cytoskeletal components responsible for the nanomechanical contraction and mechanical properties of the cell have been labeled (actin, red; α-tubulin, green). Phase-contrast, fluorescence, and nanomechanical measurements performed with a JPK Nanowizard AFM fitted with an ORCA-AG Hamamatsu deep-cooled CCD camera. Data courtesy of A.E. Pelling, B.M. Nicholls, and M.A. Horton (Department of Medicine, London Centre for Nanotechnology, UCL).

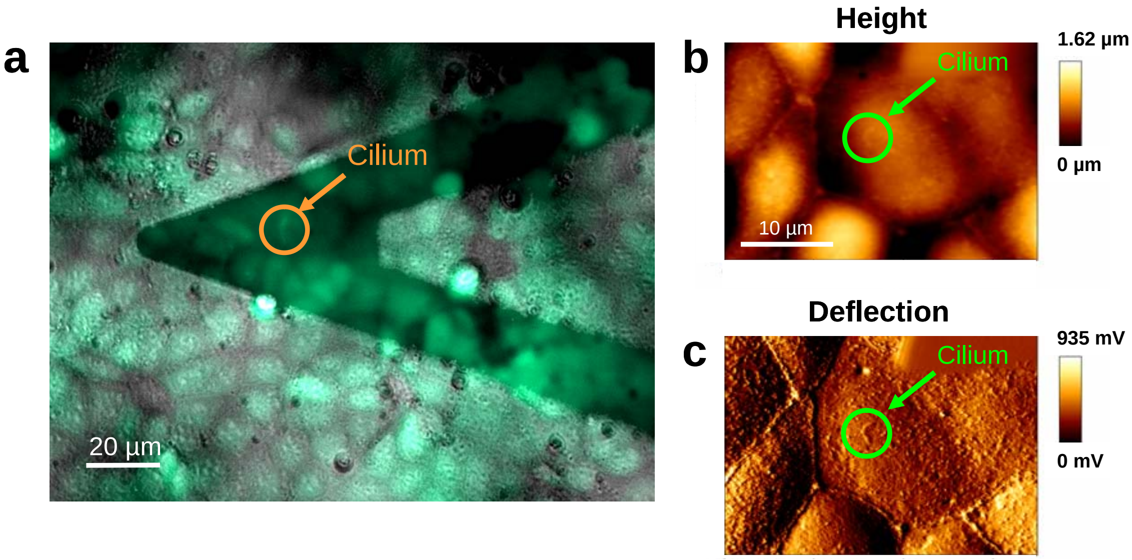

**FIGURE 4.** (a) A combined phase image and FITC image taken of AFM cantilever and primary cilia labeled (green) mouse inner medullary collecting duct (IMCD) cells. IMCD cells were fixed in 2% glutaraldehyde and imaged in PBS. Using the combined image, the AFM cantilever could be positioned to sample areas containing primary cilia (circled in orange). The image was taken using ORCA-AG Hamamatsu camera and processed using Wasabi version 1.5. (b) A contact mode height image and (c) deflection image of fixed IMCD cells using the JPK Nanowizard. A primary cilium can be seen on the centermost cell in the image (circled in green). The primary cilium had a length ~3.2 µm. The scale bars were for (a) 20 µm, (b) and (c) 10 µm. Data courtesy of B.J. Haupt, B.M. Nicholls, S. Nesbitt, and M.A. Horton (Department of Medicine, London Centre for Nanotechnology, UCL).

A little more than 20 years ago, AFM represented a paradigm shift in microscopy, away from using light or waves to visualize a surface to imaging through tactile interactions often referred to as "seeing by feeling". In their original paper [1], the inventors of the AFM predicted its utility in the measurement of small forces and mechanical properties at local scales. During the last 2 decades this has come to pass and the AFM has revolutionized mechanical studies of surfaces, single molecules and cells."

As important as this initial shift in microscopy was to the surface science and physics communities, AFM is currently undergoing a second evolution through the integration of traditional microscopies used in biological research. These new hybrid AFMs are now revolutionizing microscopy in the biomedical sciences by providing a nanomechanical “finger” that can directly probe the mechanical properties of living cells as well as actuate mechanosensitive pathways, all while allowing simultaneous measurement of signaling, gene expression, and structural remodeling. The future applications of AFM in biomedical research are continuing to expand and have not yet reached their full potential. The mix of human imagination with continuing development in new fluorescent dyes and microscopies[3,5,7,8,11, 13,18,19,26,38,40,41,42,43,55,56,57], high speed AFM imaging[65,66,67], and novel developments in AFM sensing and actuation[68,69] will likely result in several more revolutions in the biomedical and nanosciences.

## ACKNOWLEDGEMENTS

This work was supported by the IRC in Nanotechnology (AEP), EU STREP Framework 6 grant ‘Tips4Cells’ (BJH), and the Wellcome Trust (MAH). The help of JPK, Berlin in this project is acknowledged.
